# *In silico* and *in vitro* investigations of the drug–drug interaction mechanisms between fludarabine and busulfan

**DOI:** 10.3389/fphar.2026.1744021

**Published:** 2026-03-10

**Authors:** Khalil Ben Hassine, Shannon Robin, Frederic Baleydier, Mourad Mseddi, Vid Mlakar, Jayaraman Muthukumaran, Marc Ansari, Chakradhara Rao Satyanarayana Uppugunduri

**Affiliations:** 1 Cansearch Research Platform for Pediatric Oncology and Hematology, Department of Pediatrics, Gynecology, and Obstetrics, University of Geneva, Geneva, Switzerland; 2 Division of Pediatric Oncology and Hematology, Department of Women, Child, and Adolescent, University Hospital of Geneva, Geneva, Switzerland; 3 Department of Biotechnology, Sharda School of Bio-Science and Technology, Sharda University, Greater Noida, India; 4 Department of Medical Oncology, Jawaharlal Institute of Postgraduate Medical Education and Research (JIPMER), Puducherry, India

**Keywords:** drug–drug interaction, enzyme inhibition, enzyme kinetics, estimated binding affinity, glutathione-S-transferase enzyme, molecular dynamics simulation, molecular docking

## Abstract

**Background:**

Understanding drug interactions in hematopoietic stem cell transplantation (HSCT) is crucial given the narrow therapeutic index of busulfan (BU), which is a key conditioning agent. In this study, we investigate the potential impacts of fludarabine (Flu) as a frequently co-administered agent in HSCT on BU pharmacokinetics (PK). Specifically, we examine whether Flu can alter BU metabolism by affecting the predominantly expressed cytosolic glutathione-S-transferases (GSTs), particularly GSTA1, GSTM1, and GSTP1, which are essential for BU detoxification.

**Methods:**

We conducted molecular docking and atomistic molecular dynamics simulations using the crystal structures of GSTA1, GSTM1, and GSTP1 to study the estimated binding affinity of Flu as well as its metabolites to these target enzymes. We then performed *in vitro* assays on human recombinant GST enzymes and HepaRG hepatocyte cells by focusing on enzymatic inhibition, GST expression analysis, and glutathione level measurements. Enzymatic assays were conducted using 1-chloro-2,4-dinitrobenzene as a substrate alongside Western blotting and cell-viability-corrected glutathione (GSH) assays to determine the influences of Flu on the enzymatic activities and expression of GSTs.

**Results:**

*In silico* analysis predicted the binding affinities of Flu to the GST isoforms, with estimated *Ki* values of 0.2 µM, 5.2 µM, and 23.9 µM for GSTA1, GSTM1, and GSTP1, respectively; although these are micromolar (µM) inhibition values that indicate relatively weak or moderate inhibition, they are still insightful for understanding the potential interactions. However, the *in vitro* assays revealed no significant inhibition of the GST enzymes by Flu, even at concentrations up to ten times the clinical peak (C_max_). Further, Flu did not notably alter GSTA1 expression or affect cellular glutathione levels in the HepaRG cells. These experimental findings suggest that Flu may have minimal influence on the GST-mediated detoxifying pathway in the context of cancer treatment.

**Conclusion:**

The present study underscores the importance of empirically validating *in silico* observations in pharmacological research by emphasizing the minimal effect of Flu on GST activity and its implications in clinical oncology. Moreover, the findings suggest that Flu is not likely to alter BU pharmacokinetics via the GSH-conjugation pathway.

## Highlights

- Fludarabine does not inhibit GSTA1 at clinically relevant concentrations *in vitro.*
- Molecular docking and molecular dynamics simulations predict micromolar affinity of fludarabine to glutathione-S-transferase isoforms.
- Computational methods help identify potential false negatives in drug interaction studies.
- *In silico* analysis guides prioritization of drug candidates for targeted *in vitro* testing.

## 1 Introduction

Cytosolic glutathione-S-transferases (GSTs) are phase II detoxifying enzymes that are mainly implicated in cellular protection against DNA damage and oxidative stress. This protection is facilitated by the conjugation of glutathione (GSH) with reactive electrophilic compounds like anticancer drugs. Cytosolic GSTs are of seven classes, namely, alpha (α, GST-A1, A2, A3, and A4), kappa (κ, GST-K1), mu (μ, GST-M1, M2, M3, M4, and M5), omega (ω, GST-O1 and O2), pi (π, GST-P1), theta (θ, GST-T1 and T2), and zeta (ζ, GST-Z1). Several reported genetic polymorphisms exert substantial influences on GST functions and expression, resulting in various pathologies (Huezo-Diaz et al., 2014; Allocati et al., 2018).

GST enzymes are involved in the metabolism of various cytotoxic compounds, such as platinum agents (e.g., cisplatin), nitrogen mustard analogs (e.g., cyclophosphamide), and alkylating agents (e.g., busulfan (BU)) (Allocati et al., 2018; Zhong et al., 2006). Many of these compounds are widely used in adult and pediatric oncology, especially in the context of hematopoietic stem cell transplantation (HSCT), as myelosuppressive or lymphosuppressive agents (Ben Hassine et al., 2021a). The GSTA1, GSTM1, and GSTP1 isoforms are the main metabolizing enzymes of BU, which is one of the pillars of chemotherapeutic conditioning for HSCT (Huezo-Diaz et al., 2014). Czerwinski et al. (1996) showed that GSTA1 was responsible for the majority of BU metabolism, followed by GSTM1 and GSTP1. Bredschneider et al. (2002) reported similar findings by assessing human-recombinant-GST-isoform-mediated BU glutathionylation. The genetic variants affecting the expression of these isoforms (mainly GSTA1) were shown to be correlated with BU pharmacokinetics (PK), especially in terms of their individual metabolic capacities (Ben Hassine et al., 2021a; Ansari et al., 2016). However, BU exposure is correlated with transplantation-related efficacy and toxicity outcomes (Ansari et al., 2017). Evidence of direct correlation between the *GSTA1* promoter polymorphisms and treatment outcomes is also reported independent of the PK of BU (Ansari et al., 2017). In light of these known PK-outcome relationships, the drug–drug interactions (DDIs) affecting BU clearance (CL) require attention, especially in the context of pediatric HSCT, as they play pivotal roles in determining the systemic exposure to BU. Until now, very few drugs have been reported to exert pharmacokinetic DDIs with BU (Myers et al., 2017). Among these drugs, azole antifungals and metronidazole have been shown to decrease BU CL and increase systemic exposure when co-administered with either oral or intravenous BU. Despite studies demonstrating an absence of interactions for acetaminophen (Nguyen et al., 2004), co-administration with BU is not recommended given the theoretical competitive inhibition of acetaminophen’s metabolite N-acetyl-p-benzoquinone imine to GSTs (U.S. Food and Drug Administration, 2015). The mechanisms of the reported DDIs are unclear, with the most plausible hypothesis being that these agents act as GST inhibitors or deplete GSH affecting BU CL. Emerging network-based and pharmacological studies suggest that integrating the biology and genetics of GST enzymes with system-level approaches can improve prediction of DDIs with BU (Hao et al., 2021; Kim et al., 2011; Combarel et al., 2023). Fludarabine (Flu) is one of the most commonly co-administered agents with BU that is reported to reduce BU CL by approximately 6%–12% during early dosing intervals up to approximately 8%–15% on subsequent days of conditioning, with marked interindividual variability. Several researchers have observed that co-administration of Flu affects both oral and intravenous (IV) clearance of BU (de Castro et al., 2013; Nava et al., 2017; Ben Hassine et al., 2021b). Similarly, co-administration of clofarabine, which is another nucleoside analog administered along with Flu and BU, resulted in diminished BU CL (Shukla et al., 2020). Given the increasing use of regimens combining Flu and BU, it is important to assess the PK DDIs between the two agents.

Direct quantitative evidence linking reduced BU CL in the presence of Flu via GST-mediated BU metabolism is currently limited, highlighting a critical mechanistic research gap. The present study explores whether Flu has the potential to modulate GST-related pathways that could contribute mechanistically to reduced BU CL (Ben Hassine et al., 2021b). *In silico* and *in vitro* systems can help us quantify specific mechanisms even if they are minimal, which could collectively result in clinically observed reductions in BU CL. We hypothesize that Flu could decrease BU conjugation with GSH through three potential mechanisms. The first possibility is via direct inhibition of the GST enzymes (Robin et al., 2020), while the second possibility is through a decrease in GST expression and the third possibility is by decreasing the GSH reservoir in hepatic cells to diminish the cofactor and hence BU CL via GSH conjugation. The potentials of the cosolvents, i.e., dimethylacetamide (DMA) and/or its metabolite monomethylacetamide (MMA), used in the IV BU formulation to affect the enzyme activities of GSTA1, GSTM1, and GSTP1 were explored. The main objective of this work was to explore the impacts of Flu on the enzyme activities of GSTA1, GSTM1, and GSTP1 through a combination of *in silico* and *in vitro* studies.

## 2 Methods

### 2.1 Materials

The human recombinant GST enzymes GSTA1, GSTM1, and GSTP1 were purchased from MyBioSource (San Diego, CA, United States), aliquoted, and stored at −80 °C. Ethacrynic acid (EA), Flu, BU, 1-chloro-2,4-dinitrobenzene, DMA, MMA, GSH, phosphate-buffered saline (PBS), ethanol, and dimethyl sulfoxide (DMSO) were purchased from Sigma-Aldrich (St. Louis, MO, United States). Stock solutions of Flu (100 mM), EA (25 mM), and BU (200 mM) were prepared in DMSO, while stock solutions of CDNB (100 mM) were prepared in 95% ethanol and stock solutions of GSH (25 mM) were prepared in PBS. Each stock solution was aliquoted as needed and stored at −20 °C. HepaRG cells, William’s E medium, HepaRG thawing/plating/general-purpose supplement, metabolism and growth supplement, RIPA lysis and extraction buffer, protease and phosphatase inhibitor cocktails, and BCA protein assay were purchased from Thermo Fisher Scientific (Waltham, MA, United States). The DNeasy Blood and Tissue kit for DNA extraction was purchased from Qiagen (Hilden, Germany), while GSH-Glo and CellTiter-Glo assay kits were purchased from Promega (Madison, WI, United States). The murine anti-M1 antibody was purchased from Thermo Fisher Scientific, while rabbit anti-A1, anti-P1, and anti-T1 monoclonal antibodies were purchased from Abcam (Cambridge, United Kingdom). Goat anti-mouse horseradish peroxidase (HRP) and anti-rabbit HRP antibodies were purchased from Bio-Rad (Hercules, CA, United States).

#### 2.2 Molecular docking and molecular dynamics (MD) simulations

The crystal structures of human GSTA1, GSTM1, and GSTP1 isoforms were retrieved from the Research Collaboratory for Structural Bioinformatics Protein Data Bank (RCSB PDB; URL: www.rcsb.org) (Burley et al., 2017) and chosen according the following criteria: resolution of the crystal structure, Ramachandran or phi–psi angle, wwPDB quality evaluation, and presence/absence of GSH in the crystal structure. The Simplified Molecular Input Line Entry System (SMILES) notation of each studied ligand was obtained from the NCBI PubChem database (https://pubchem.ncbi.nlm.nih.gov/), while the three-dimensional XYZ or Cartesian coordinates (or PDB file) were generated from SMILES using the CORINA 3D structure conversion web server (https://mn-am.com/products/corina/). The ligands tested by molecular docking included EA and curcumin (positive controls), Flu and its triphosphorylated metabolite Flu-ATP, BU and its metabolite tetrahydrothiophene, sulfolane and sulfolane-3-OH, and treosulfan and its active epoxy forms EBDM and DEB. The protein and ligand 3D structures were then prepared by adding polar hydrogen as well as merging non-polar hydrogens and partial charges (ligands: Gasteiger partial charges and Proteins: AMBER-derived Kollmann charges) using Molecular Graphics Laboratory (MGL) Tools (version 1.5.7) (Morris et al., 2009) before exporting to the PDBQT (XYZ coordinates (PDB) + partial charges (Q) + AutoDock Atom types (T) format. To find all possible ligand-binding sites on the target proteins, we performed both blind or non-specific as well as direct or site-specific molecular docking calculations. Site-specific molecular docking restricts the binding grid box coordinates to the ligand-specific binding sites of the protein, particularly the H-site of the target enzyme. To comprehensively explore all potential ligand-binding sites during molecular docking, the exhaustiveness parameter was set to 8 and energy range was fixed at 3. The identification of the binding pocket for each isoform was based on literature describing the binding sites of the GSTs and/or extracted from the UniProtKB (UniProt Knowledgebase) database (Universal Protein Resource; www.uniprot.org; UniProt Consortium, 2019). If available, the chosen three-dimensional protein structures were superimposed with a ligand-bound structure of the same enzyme from the PDB database. Molecular docking was performed in the presence or absence of the cofactor GSH. Both site-specific and blind docking calculations were performed using PyRx software (version 0.8; The Scripps Research Institute, USA) running the AutoDock Vina computation tool (Trott and Olson, 2010). AutoDock Vina generates nine best docking poses for each ligand with the binding site of each enzyme structure. The complex with the lowest free binding energy (*ΔG* in kcal/mol) was selected for further atomistic MD simulations. The analysis of the protein–ligand molecular interactions of the docked structures was performed using the Protein Ligand Interaction Profiler online tool (PLIP; https://plip-tool.biotec.tu-dresden.de/plip-web/plip/index) (Adasme et al., 2021). MD simulations of the resulting complexes of EA and Flu with GSTA1, GSTM1, and GSTP1, along with the unbound forms, were performed using GROMACS version 2020 (Van Der Spoel et al., 2005; Abraham et al., 2015). The detailed protocols and steps involved in the MD simulations are described elsewhere (Ben Hassine et al., 2022; Robin et al., 2022). Following completion of the atomistic MD simulations, trajectory corrections were performed for all systems using the *gmx trjconv* command with the flags –center and –pbcnojump to eliminate periodic boundary artifacts and recenter the system. Subsequently, the corrected trajectories were subjected to detailed structural analyses using various built-in GROMACS tools, including root mean-squared deviation (RMSD; *gmx rms*), root mean-squared fluctuation (RMSF; *gmx rmsf*), radius of gyration (Rg; *gmx gyrate*), and solvent-accessible surface area (SASA; *gmx sas*). Apart from the abovementioned global dynamics analyses, the essential dynamics of proteins and protein–ligand complexes were analyzed using two GROMACS commands, namely, *gmx covar* and *gmx anaeig* (Van Der Spoel et al., 2005; Kumari et al., 2014). This analysis plays a crucial role in identifying biologically meaningful, collective, and dominant motions and is fundamentally a linear data reduction technique based on principal component analysis (PCA).

### 2.3 *In vitro* assays

#### 2.3.1 HepaRG cell culture, DNA, and protein extraction

HepaRG cells were grown in a medium supplemented with thawing/plating/general-purpose supplement until confluent. Thereafter, the cells were resuspended in a medium supplemented with metabolism and growth supplements and seeded in 96-well plates at 10,000 cells per well before incubation for 24 h before treatment. Total protein extraction was performed on pellets collected from confluent 25-cm^2^ flasks using RIPA lysis buffer containing phosphatase and protease inhibitors following a standard protein extraction protocol. The protein quantification was performed using the BCA kit according to manufacturer protocols. The DNA extraction was performed using the DNeasy Blood and Tissue kit according to manufacturer protocols, and DNA quantification was performed using a NanoDrop spectrophotometer (Thermo Fisher Scientific) at 260 nm. The excitation/emission ratio of 260/280 nm was also assessed as a quality indicator for the extraction.

#### 2.3.2 Enzymatic assays with recombinant human GSTs

The DNA obtained from HepaRG cells was used for genotyping the GSTA1 promoter region variants and for GSTM1 null status using Sanger sequencing and melt curve analysis, respectively. In this study, CDNB was used as a surrogate substrate because it is a well-validated pan-GST probe that strongly reports GSH-dependent conjugation activities across major cytosolic GST isoforms relevant to BU metabolism, including GSTA1, GSTM1, and GSTP1. Its high and reproducible catalytic turnover makes it a sensitive indicator of functional GST modulation at the enzyme level. The GST enzyme assay using recombinant GST proteins was performed as described previously by our group (Robin et al., 2020; Robin et al., 2022). In brief, CDNB (0.25–1.5 mM) was used as the substrate with 0.05 mg/mL of the recombinant GST proteins (or 0.5 mg/mL of HepaRG-extracted proteins) and 2.5 mM of GSH as the cofactor. Enzymatic reaction was initiated upon addition of the substrate, and a blank without the added enzyme was used to correct for the spontaneous conjugation of CDNB with GSH. For reactions with inhibitors, the protein, cofactor, and inhibitor were incubated for 5 min before addition of the substrate. EA was used as the positive control for the inhibition assays, while Flu (F-ARA-A), DMA, and MMA were used as the test molecules. The ethanol concentration in the reaction mixture was 5%, while the DMSO concentration was 1%. These solvent concentrations do not influence the *in vitro* activities of the GSTs (Robin et al., 2020). We used the same protocol to test the GST enzyme activities of the protein extracts from HepaRG cells using 0.5 mg/mL of each extract. The K_m_ and V_max_ values of CDNB with the different recombinant GST proteins were determined using five concentrations of CDNB. The experimentally determined K_m_ was used as the substrate concentration in the inhibition assays. For inhibition evaluation, a preliminary screening was performed using three concentrations of putative inhibitors based on their clinically observed peak concentrations (C_max_, 5C_max_, and 10C_max_). If enzyme inhibition was observed in this concentration range, only then was it defined for screening the IC_50_ and inhibition constant *K*_*i*_. The IC_50_ value was calculated using a single concentration of the substrate and seven concentrations of the inhibitor (EA at 1, 2.5, 5, 10, 25, 50, and 100 µM) with a range based on the results of the preliminary experiments. Each experiment was performed in triplicate to ensure reliability.

#### 2.3.3 Characterization of GST genotypes and their expression in HepaRG cells

*GSTA1* haplotypes in the HepaRG cells were genotyped using a long-range polymerase chain reaction (PCR) and Sanger sequencing method described previously (Mlakar V. et al., 2021). *GSTM1* and *GSTT1* gene deletions were screened using a previously described real-time PCR method with multiplex amplification (Mlakar SJ. et al., 2021). *GSTP1* haplotypes were screened using an allele-specific PCR method described earlier (Ansari et al., 2017). To assess whether Flu inhibits *GSTA1* expression in the HepaRG cells, we used Western blots to compare *GSTA1* expression in the untreated cells and cells treated with Flu at 40 µM for 24 h. The Western blotting protocol for the GSTs is as described in an earlier work (Mlakar SJ. et al., 2021).

#### 2.3.4 GSH levels and viability assays

The cells were plated at the rate of 10,000 cells (HepaRG) per well in a 96-well plate and incubated for 3 h, 24 h, and 72 h in William’s E medium with the HepaRG maintenance and metabolism supplement. EA was used as a positive control for GSH depletion in the cells, while Flu and BU were tested as independent treatments and as co-treatments in the cells. The concentrations of the compounds tested were based on maximum concentrations (C_max_) in children and adults (if pediatric data were unavailable). The concentrations tested for each molecule were equivalent to C_max_ and 10 times of C_max_ (Molnar and Somberg, 2009; Chung et al., 2019). The GSH and cell viability assays were performed in parallel for each tested condition. The GSH levels were measured using the GSH-Glo Kit according to the manufacturer’s protocols. Cell viability was measured using the CellTiter-Glo kit according to the manufacturer’s protocol. The luminescence was measured using a SpectraMax iD3 microplate reader (Molecular Biosciences, San Jose, CA, United States). The viability-adjusted GSH levels were then calculated for each experimental condition. The experiments were conducted in triplicates. The protocols used to determine the K_m_ and V_max_ values are provided in the Supplementary Material.

## 3 Results

### 3.1 Molecular docking and MD simulation

**FIGURE 1:**
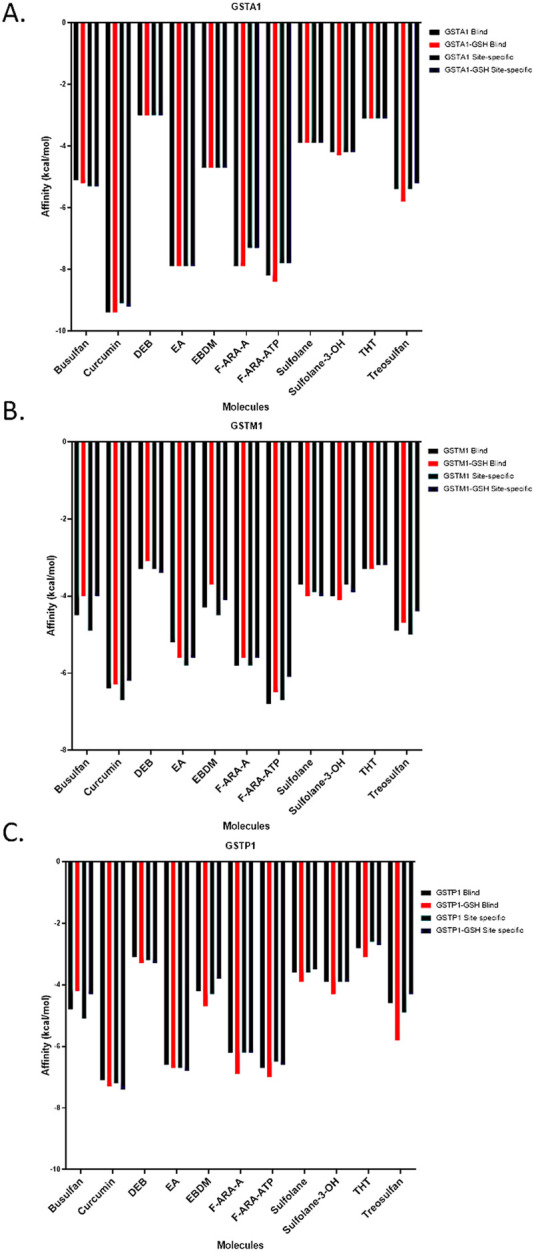
Estimated binding affinities of **(A)** GSTA1, **(B)** GSTM1, and **(C)** GSTP1 with various molecules computed via direct and blind docking calculations. Direct docking was performed at the known hydrophobic site, whereas blind docking explored potential binding regions across the entire protein surface.

The PDB identifiers or accession numbers of the protein crystal structures chosen for molecular docking and MD simulations are 6ATO (1.55 Å) for GSTA1, 1XW6 (1.90 Å) for GSTM1, and 5GSS (1.95 Å) for GSTP1. The results of the blind and site-specific molecular docking are presented in Figure 1. The docked positions of Flu as well as positive controls curcumin and EA with the selected GST enzymes are shown in Supplementary Figure S1 and Table 2. The predicted binding free energy (*ΔG*) of Flu was similar to those of curcumin and EA for the three GST isoforms, suggesting that Flu could bind to the active sites of all three GST enzymes. The triphosphate form of Flu was also predicted to have a similar binding affinity with the active sites of all three tested GST enzymes, similar to those of Flu, EA, and curcumin. The *ΔG* of BU was lower than those of the positive controls, and the downstream metabolites of BU showed estimated binding free energies akin to those of their parent compound. Treosulfan had a similar binding affinity as BU, probably because of its similar scaffold or structural backbone. Regarding the binding site residues of GSTA1 with the different ligands, the tested compounds shared intermolecular H-bonds with the amino acid residues arginine 13 and serine 18; curcumin and EA shared hydrophobic interactions with residues leucine 107 and tyrosine 166 of GSTA1. These hydrophobic interactions were not predicted with Flu and its triphosphorylated form. Similarly, no intermolecular hydrophobic interactions were predicted for Flu and its triphosphate form with the other GST isoforms, while a few hydrogen bonds were common between these compounds and the positive controls (Table 1).

**TABLE 1:** Intermolecular non-covalent interaction profiles and interacting residues of the target enzymes GSTA1, GSTM1, and GSTP1 with two positive controls (ethacrynic acid and curcumin) and the lead molecules (fludarabine and its derivatives).

GST isoform	Substrate residue interactions (from literature)	Type of interaction	Residue	Amino acid	Ethacrynic acid (reference)	Curcumin	Fludarabine	Fludarabine triphosphate
GSTA1	10, 14, 107, 108, 111, 166, 208, 222	Hydrophobic interaction	106	ILE	•	​	​	​
​	​	Hydrophobic interaction	107	LEU	•	•	​	​
​	​	Hydrophobic interaction	107	LEU	•	​	​	​
​	​	Hydrophobic interaction	166	TYR	•	•	Hydrogen bond	​
​	​	Hydrophobic interaction	166	TYR	•	​	​	​
​	​	Hydrophobic interaction	166	TYR	•	​	​	​
​	​	Hydrogen bond	13	ARG	•	•	•	•
​	​	Hydrogen bond	15	ARG	•	​	​	​
​	​	Hydrogen bond	17	GLU	•	​	​	​
​	​	Hydrogen bond	18	SER	•	•	•	•
​	​	Hydrogen bond	169	GLU	•	​	​	​
​	​	π-stacking	166	TYR	•	​	​	​
GSTM1	6, 7, 9, 12, 111, 115, 208, 209	Hydrophobic interaction	6	TYR	•	​	​	​
​	​	Hydrophobic interaction	115	TYR	•	•	​	​
​	​	Hydrophobic interaction	115	TYR	•	π-stacking	π-stacking	​
​	​	Hydrogen bond	6	TYR	•	​	•	​
​	​	Hydrogen bond	7	TRP	•	•	​	​
​	​	Hydrogen bond	42	ARG	•	​	​	​
​	​	Hydrogen bond	45	TRP	•	​	​	​
​	​	Hydrogen bond	59	LEU	•	​	​	​
​	​	Hydrogen bond	111	GLY	•	•	​	•
​	​	Hydrogen bond	115	TYR	•	​	•	•
​	​	Hydrogen bond	115	TYR	•	​	​	​
​	​	Salt bridge	49	LYS	•	​	​	​
GSTP1	7, 8, 10, 13, 104, 108, 204, 205	Hydrophobic interaction	8	PHE	•	•	​	​
​	​	Hydrophobic interaction	8	PHE	•	•	​	​
​	​	Hydrophobic interaction	9	PRO	•	​	​	​
​	​	Hydrophobic interaction	10	VAL	•	•	​	​
​	​	Hydrophobic interaction	35	VAL	•	​	​	​
​	​	Hydrogen bond	7	TYR	•	•	•	​
​	​	Hydrogen bond	13	ARG	•	​	•	​
​	​	Hydrogen bond	35	VAL	•	​	​	​
​	​	Hydrogen bond	108	TYR	•	•	•	​
​	​	Hydrogen bond	204	ASN	•	​	​	​
​	​	Hydrogen bond	205	GLY	•	​	​	•

**FIGURE 2:**
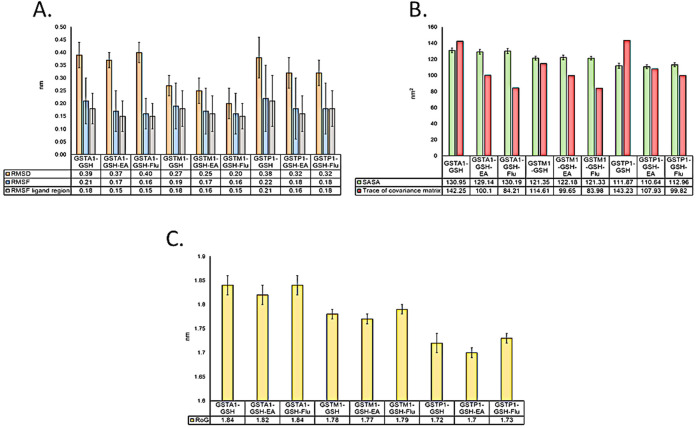
Comparative analyses of the global dynamic parameters of GSTA1, GSTM1, and GSTP1 complexed with different ligands: **(A)** structural stability parameters as well as **(B, C)** compactness and overall atomic motion parameters are represented by the root mean-squared deviation (RMSD), root mean-squared fluctuation (RMSF), solvent-accessible surface area (SASA), and radius of gyration (Rg).

The MD simulation results are shown in Supplementary Figure S2. The stabilities of the GST-ligand complexes were analyzed using various structural metrics, including RMSD and RMSF, as summarized in Figure 2. The shape, compactness, and folding properties of the unbound and ligand-bound forms of the GSTs were analyzed through their Rg measurements. Similarly, the SASA analysis revealed conformational changes in the GSTs upon ligand binding. The combined results from the global analyses (RMSD, RMSF, Rg, and SASA) and essential dynamics analysis (PCA) show that Flu forms stable complexes with all GST enzymes, similar to the positive control EA. Thus, the *in silico* exploration results indicate that Flu could be a ligand of the GST enzymes and could cause competitive inhibition of these enzymes, with predicted *K*_*i*_ value of 0.2 µM, 5.2 µM, and 23.9 µM for GSTA1, GSTM1, and GSTP1, respectively.

### 3.2 *In vitro* investigation

#### 3.2.1 Enzymatic assays for GST inhibition

HepaRG cells were found to carry the GSTA1 rapid metabolizer diplotype and not the GSTM1 null allele (Supplementary Figures S3A, B). Hence, the *in vitro* experiments must be observed in the light of the functional GSTA1 and GSTM1 in HepaRG cells (Supplementary Figures S3A, B). The results of the enzymatic kinetic assays are presented in Table 1. The Michaelis constant (K_m_) of CDNB was determined for each isoform and used in the corresponding inhibition assay. The K_m_ value of CDNB was the lowest for GSTP1 and highest for GSTM1. The K_m_ of CDNB with GSTA1 (0.61 ± 0.06 mM) was lower than the previously observed value for BU (3.74 ± 0.88 mM) (Bredschneider et al., 2002), suggesting that CDNB has more affinity toward the active site of GSTA1 compared to BU. The V_max_ and K_cat_ values indicate that GSTA1 has the highest activity for GSH conjugation of CDNB, followed by GSTM1 and GSTP1. This finding is consistent with the relative contributions of these three enzymes to the GSH conjugation of BU. However, the V_max_ values are approximately 7,000-fold higher for the metabolism of CDNB by each isoform compared to BU. Together, these results suggest that the relative contributions of the three tested isoforms are similar between BU and CDNB, making CDNB an acceptable probe for GST activity in place of BU. However, the fact that CDNB has a greater affinity for these enzymes and that their V_max_ values for CDNB conjugation are much higher than those for BU must be considered carefully (Supplementary Material). Accordingly, the K_m_ value of CDNB for each isoform was used in further inhibition experiments. The IC_50_ and *Ki* values were determined for the positive control EA, which were least for GSTM1, followed by GSTP1 and GSTA1 (Table 1; Figure 1). However, for Flu, concentrations up to 10 times the clinically observed C_max_ did not inhibit any of the GST isoforms tested herein. Similar results were obtained with the proteins extracted from the HepaRG cells (Supplementary Figure S4). These suggest that Flu is unlikely to inhibit GSTs *in vivo* at the tested concentrations. Furthermore, DMA and MMA did not inhibit the tested recombinant GST isoforms (Table 2). These results rule out the hypothesis that Flu interacts with BU via GST inhibition.

**TABLE 2:** Kinetic parameters of the GST isoenzymes (GSTA1, GSTM1, and GSTP1) and comparative inhibitory profiles of the control and lead compounds.

Enzyme kinetic parameter		GSTA1	GSTM1	GSTP1
K_m_CDNB_ (mM)		0.61 ± 0.06	0.81 ± 0.19	0.45 ± 0.09
V_max_CDNB_ (μM/min)		382.2 ± 11	20.68 ± 2.23	9.54 ± 0.74
K_cat_ (s^-1^)		34.85 ± 2.72	1.8 ± 0.1	0.73 ± 0.1
EA (positive control)	*Kd: in silico* prediction (µM)	1.0	47.1	12.2
	IC_50_ (µM)	9.62 ± 1.08	0.33 ± 1.29	3.75 ± 1.28
	K_i_ (µM)	21.83	0.24	3.10
	Mode of inhibition	Competitive	Competitive	Competitive
Fludarabine (F-ARA-A)	*Ki: in silico* prediction (µM)	0.2	5.2	23.9
	Preliminary *in* *vitro* inhibition (µM)	No inhibitions at 5, 25, and 50 µM*	No inhibitions at 5, 25, and 50 µM*	No inhibitions at 5, 25, and 50 µM*
DMA	*Ki: in silico* prediction (µM)	1,632.4	2,288.0	3,208.4
	Preliminary *in* *vitro* inhibition (µM)	No inhibition at 60 µM*	No inhibition at 60 µM*	No inhibition at 60 µM*
MMA	*Ki: in silico* prediction (µM)	2,709.0	3,208.3	2,709.1
	Preliminary experiment (µM)	No inhibition at 60 µM*	No inhibition at 60 µM*	No inhibition at 60 µM*

The kinetic parameters (K_m_, V_max_, and K_cat_) were determined using CDNB as the substrate. The control compound ethacrynic acid (EA) and lead molecule fludarabine (F-ARA-A), along with its derivatives dimethylacetamide (DMA) and monomethylacetamide (MMA), were evaluated using *in silico* binding affinity predictions (K_d_, K_i_) as well as preliminary *in vitro* inhibition assays (IC_50_). All three GST isoenzymes exhibited competitive inhibition with EA, whereas fludarabine and its derivatives showed no significant inhibitions at the indicated concentrations (up to 50–60 µM). EA was tested at concentrations of 0, 1, 2.5, 5, 10, 25, 50, and 100 µM.

#### 3.2.2 Fludarabine effects on GST expression and GSH levels

**FIGURE 3:**
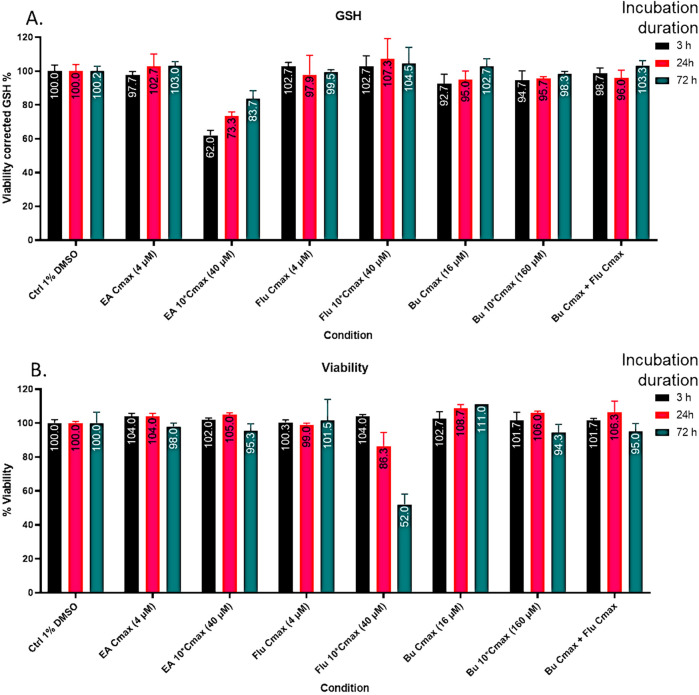
**(A)** Viability-corrected glutathione (GSH) levels in the HepaRG cells were determined using the maximum clinical concentration (C_max_) and multiples of C_max_ with the positive control ethacrynic acid (EA) and lead molecule fludarabine (Flu). **(B)** Decreases in the viability-corrected GSH levels in the cells at 3, 24, and 72 h post-treatment.

Because the action mechanism of Flu involves inhibition of DNA and protein synthesis, we evaluated its potential for reducing GSTA1 expression. As shown in Supplementary Figure S4, Western blotting analyses of the proteins extracted from cells incubated with Flu at C_max_ did not show altered expression of GSTA1. The viability-corrected GSH levels of the HepaRG cells were not altered by Flu when tested at concentrations up to 10C_max_ (Figure 3). In contrast, EA decreased GSH levels by 38% (cell viability-corrected) at 40 µM (10 times its C_max_ observed in adult patients; Molnar and Somberg, 2009) 3 h post treatment of the cells. Incomplete recovery of the GSH level was observed 72 h post-exposure, and increases in the GSH levels were observed at 24-h and 72-h post-treatment with EA (Figure 3).

## 4 Discussion

In this study, we explored the influences of Flu on BU glutathionylation catalyzed by GSTM1, GSTP1, and GSTA1. We explored the impacts of these interactions by assessing GST enzyme activities and protein expression as well as cellular levels of GSH in the presence and absence of Flu. Although the *in silico* explorations indicated comparable binding affinities between Flu and all GST isoforms tested herein with those of the positive control EA, the *in vitro* inhibition assays did not corroborate the *in silico* predictions. In this study, we expected the molecular docking and MD simulations to align with the *in vitro* results. *In silico* observations on the higher affinities of Flu with the hydrophobic sites of GSTM1, GSTP1, and GSTA1 were comparable to those of the GST competitive inhibitor, suggesting competitive inhibition of the GSTs by Flu. However, differences were observed in the binding patterns between the positive control EA and Flu in terms of molecular interactions (Table 1), which may explain the non-concordance between the *in silico* predictions and *in vitro* observations. Curcumin and EA are both known to be GST inhibitors (Robin et al., 2020) that were predicted to form hydrogen bonds with the active-site residues but not Flu. Herein, atomistic MD simulations were used to complement the *in vitro* enzymatic assays but could not fully replace them for evaluating potential DDIs. Although MD and other *in silico* methods offer valuable mechanistic insights and help prioritize candidate molecules, they may yield false positives owing to limitations in modeling biological complexity. However, these approaches remain helpful for minimizing false negatives and narrowing large compound libraries. It is also possible that Flu could inhibit the GST isoforms at concentrations beyond those tested here; however, such effects are unlikely to be clinically significant, as our experiments included doses up to ten times the peak plasma concentrations typically employed in clinical settings.

MD simulation results should be carefully interpreted and further confirmed with *in vitro* assays. In this study, we could not demonstrate the usefulness of molecular docking and MD simulations for predicting competitive DDIs. Furthermore, MD analyses require very high computing power but produce low throughput owing to the lengthy simulation times. Alternative molecular docking algorithms, such as the one implemented in the MORDOR program, can account for target structure flexibility and may be employed for timely screening of ligand libraries for the target binding affinities (Tessaro and Scapozza, 2020). Although our molecular docking and MD simulations offer valuable preliminary insights into the interactions between Flu and the GST isoforms, the *in silico* results could not be verified with *in vitro* testing. Here, we show that Flu does not influence the glutathionylation pathway by inhibiting GST enzyme activity, thus ruling out our hypothesis. Moreover, Flu did not affect the expression or protein or cofactor (e.g., GSH) levels, ruling out the possibility of inhibiting glutathionylation of BU in the presence of Flu. An alternative hypothesis for the DDIs of Flu with BU as proposed by other researchers is that CYP activity modulators (inhibitors or inducers) may affect BU CL given the fact that several CYPs and FMO3 are involved in the oxidation of downstream metabolites, such as tetrahydrothiophene (THT), sulfolane, and sulfolane-3-OH (Huezo-Diaz et al., 2014; El-Serafi et al., 2017). However, oxidation of the downstream metabolites of BU shall not be considered as BU CL. From a PK perspective, BU CL only accounts for the disappearance of the parent compound from systemic circulation; this does not include transformation of the downstream metabolites but includes BU metabolism to the downstream metabolites (metabolic clearance) and excretion of the unchanged form, although the latter is insignificant (Lawson et al., 2021). Because of its alkylating property, the proportion of BU that irreversibly alkylates DNA also disappears from systemic circulation and can be accounted for by the total clearance of BU. The *in vivo* amount of BU alkylation remains to be clearly determined. The spontaneous hydrolysis of BU is also part of its elimination process (Huezo-Diaz et al., 2014). Nevertheless, El-Serafi et al. (2017) showed that a feedback loop might exist, with increasing amounts of THT altering BU CL in a mouse model. As BU is not directly oxidized, this could be a valid explanation for the effects of CYP and FMO activity modulators on BU CL, but the influences of Flu on CYP and FMO enzymes need to be explored.

The discrepancy between the *in silico* calculations and *in vitro* GST inhibition by Flu can be attributed to the limitations of the methodology employed in the molecular docking and MD simulations. GSTs are bisubstrate enzymes with distinct G-site (GSH-binding) and H-site (hydrophobic substrate-binding) regions, and favorable molecular docking at one site may not necessarily translate to effective inhibition of CDNB or GSH-dependent catalytic turnover, particularly in multi-isoform GST assays. Molecular docking employs simplified scoring functions and static protein conformations, limiting its ability to capture complete enzyme dynamics, solvation, entropic effects, and co-substrate involvement critical for GST catalysis. Extensive endpoint binding free energy approaches, such as molecular mechanics/Poisson–Boltzmann surface area (MM/PBSA), molecular mechanics/generalized Born surface area (MM/GBSA), and alchemical methods like free energy perturbation (FEP) and thermodynamic integration (TI), could provide additional insights prior to *in vitro* testing. Additionally, CDNB is known to exhibit GST-isoform-dependent affinity, which may further contribute to the observed divergence between computational and experimental observations. *In vitro* experiments provide evidence for similarities between BU and CDNB–GSH conjugation in terms of the relative contributions of GSTM1, GSTA1, and GSTP1 to the conjugation of the two compounds, suggesting that CDNB could be an alternative probe for evaluating the impacts of the GST isoforms on BU–GSH conjugation. However, CDNB exhibits greater affinity for GSTs than BU, and the V_max_ values of these enzymes for CDNB conjugation are much higher than those of BU. This needs to be considered carefully while interpreting the observations. Hence, K_m_ values for CDNB specific to each GST isoform were used in our inhibition experiments. We studied GSTA1 expression because it is a predominantly expressed hepatic GST isoform and major contributor to BU CL. The other isoforms like GSTM1 and GSTP1 also exhibit substantial genetic and interindividual variability. Hence, comprehensive protein-level profiling of all GST isoforms will be conducted in the future.

Pediatric HSCT studies employing Flu–BU conditioning regimens report that Flu (measured in terms of its active metabolite 2-F-ara-A) reaches peak plasma concentrations (C_max_) of approximately 0.8–1.8 mg/L (≈3–7 µM) typically at or shortly after completion of IV Flu infusion and overlap with BU administration during conditioning. PK analyses further indicate that Flu exhibits low plasma protein binding, with an unbound fraction of approximately 85%–90%, suggesting that the total plasma concentration closely reflects the pharmacologically relevant free drug level. These clinically observed concentrations fall within the lower range of those tested in the *in vitro* GST inhibition assays and are comparable to the predicted *in silico Ki* values, thereby supporting the translational relevance of the *in vitro* experimental design (Bonin et al., 2007; Nath et al., 2024). However, direct inhibition of the GST-isoform-mediated BU CL *in vitro* by Flu may provide clear insights, although CDNB is widely used as the substrate for assessing GST activity.

Considering the observations of this study, we propose that extensive target-fishing approaches, including chemoproteomics, network pharmacology, and network toxicology, coupled with experimental validation are required to elucidate the alternative mechanisms underlying Flu activity. Such strategies would enable identification of off-target interactions, upstream regulatory proteins, and pathway-level effects that could contribute to the reduced BU CL observed in clinics.

## 5 Conclusion

The precise mechanisms underlying Flu-associated reduction in BU clearance remain unresolved. However, our findings suggest that modulation of GST activity is unlikely to be the primary driver of the observed DDIs between Flu and BU. Importantly, the present study underscores the need to validate *in silico* predictions experimentally and highlights the importance of future investigations to elucidate the alternative mechanistic pathways.

## Data Availability

The original contributions presented in the study are included in the article/Supplementary Material, further inquiries can be directed to the corresponding author/s.

## Supplementary material

SUPPLEMENTARY FIGURE S1 Illustration of various intermolecular non-covalent interactions observed from the molecular docking studies of GSTA1, M1, and P1 enzymes with lead candidates. The interaction profiles were generated using the Protein–Ligand Interaction Profiler (PLIP). The interaction visualizations were further refined and rendered using PyMOL.

SUPPLEMENTARY FIGURE S2 Global dynamic and essential structural attributes derived from the molecular dynamics (MD) simulations of both unbound and ligand-bound GST enzymes. The analyses were performed using GROMACS, and the corresponding plots were generated using XMGrace. The evaluated parameters include root mean-squared deviation (RMSD) for assessing the overall structural stability, root mean-squared fluctuation (RMSF) for analyzing the residue-level flexibility, and principal component analysis (PCA) for capturing the collective motions and essential dynamic features of the protein–ligand complexes.

SUPPLEMENTARY FIGURE S3 HepaRG genotyping results for GSTA1 and GSTM1: **(A)** electropherograms of the promoter region variants of GSTA1 defining the metabolizer diplotypes; **(B)** melt curve analyses of GSTM1.

SUPPLEMENTARY FIGURE S4
**(A)** GSTA1 protein expression results from the HepaRG cells. **(B)** Total protein GST activity enzyme kinetics parameters in the presence and absence of the putative inhibitor (Flu) and positive control (EA).
