# Supplementary material for: In silico and in vitro investigations of the drug–drug interaction mechanisms between fludarabine and busulfan
Source: Front Pharmacol. 2026 Mar 10;17:1744021. doi: 10.3389/fphar.2026.1744021 (PMC13010162; doi:10.3389/fphar.2026.1744021)
Supplement: Supplementary file 1 [file Supplementaryfile1.docx]

**Supplementary Data**

1. **Enzymatic Activity and Kinetic Analysis of Human Glutathione S-Transferase Isoforms**

**Material**

- L-glutathione reduced / GSH (Sigma, ref. G4251) at 25 mM

Molecular weight: 307.5 g/mol

Prepare the GSH by diluting the powder in Millipore water.

- 1-chloro-2.4-dinitrobenzene / CDNB (Sigma, ref. 237329) at 100 mM

Molecular weight: 202.55 g/mol

Prepare the solution by diluting the CDNB powder in 95% ethanol to have a stock solution at 100 mM.

- Dulbecco’s phosphate buffered saline pH 7.1 / PBS (Sigma, ref.D8537)
- Recombinant Human glutathione S-transferase alpha-1 (MyBioSource, MBS203481) and human glutathione s-transferase pi-1 (MBS2097250)
- Millipore water
- Ethanol 99%

**Protocol**

1. Prepare five different concentrations of CDNB.

1. Prepare the assay solution.

| **Reagent** | **1 well** | **50 wells** | **100 wells** |
| --- | --- | --- | --- |
| GSH 25 mM | 10 ul | 500 ul | 1000 ul |
| PBS | 75 ul | 3.75 mL | 7.5 ml |
| Total volume | 85 ul | 4.25 mL | 8.5 ml |

1. Prepare the enzymatic solution at 0.05 mg/ml in water.

| **GST isoform** | **Stock (mg/ml)** | **Final concentration**  **(mg/ml)** | **Dilution** | **Volume GSTA1 (ul)** | **Volume water (ul)** | **Final volume (ul)** |
| --- | --- | --- | --- | --- | --- | --- |
| GSTA1 |  | 0.05 |  |  |  | 180 |
| GSTM1 |  | 0.05 |  |  |  | 180 |
| GSTP1 |  | 0.05 |  |  |  | 180 |
| GSTT1 |  | 0.05 |  |  |  | 180 |

In a 96-well plate, put 10 ul of the enzyme solution at 0.05 mg/ml or 10 ul of water for the blank wells.

|  | **1** | **2** | **3** | **4** | **5** | **6** |
| --- | --- | --- | --- | --- | --- | --- |
| **A** | **CDNB 0.25mM** | | | **Blank CDNB 0.25mM** | | |
| **B** | **CDNB 0.5mM** | | | **Blank CDNB 0.5mM** | | |
| **C** | **CDNB 1mM** | | | **Blank CDNB 1mM** | | |
| **D** | **CDNB 3 mM** | | | **Blank CDNB 3 mM** | | |
| **E** | **CDNB 5 mM** | | | **Blank CDNB 5 mM** | | |

1. Add 85 ul of the assay solution.
2. Add 5ul of the corresponding CDNB concentration, in the test and the blank wells.
3. Record the absorbance at 340 nm every minute for ten minutes.
4. Calculate the absorbance per minute.
5. Blank-correct the results.
6. According to the measured values, calculate the velocity of the reaction using equation 1.

**Equation 1.**

$$velocity \left( \frac{\mu mol}{mL}/\min or mM/min \right)=\frac{\frac{A340}{min}*V_{total}}{\varepsilon_{GS-DNB}*V_{enzyme}}$$

where A340/min is the experimentally determined change in absorbance per minute, V_total_(total volume) equals 0.2 mL, V_enzyme_(volume of enzyme) is 0.02 mL, and ε_GS-DNB_ is the molar extinction coefficient of the GS-DNB conjugate at 340 nm (9.6 μM^-4^*cm^-1^). In a 100 μL well of a 96-well plate, the path length is 0.29 cm and the extinction coefficient equals 2.784 μM^-1^. The velocity can be represented either by μmol/mL/min or mM/min.

1. Perform the analysis of the Michaelis-Menten constant with GraphPad Prism.
   1. In the columns, put the CDNB concentration in mM.
   2. In the rows, put the triplicate of the velocity and according to the correct CDNB concentration, calculated during the previous step.
   3. Analyze > non-linear regression > Enzyme kinetics – velocity as a function of substrate >Michaelis-Menten.
   4. You will obtain a graph, and a table, which resume the determined values of Vmax and Km. Check that the R square is more than 0.95. If yes, the results are accepted. Otherwise, repeat the experiment.
2. In parallel, draw the Hanes-Wolff plot with GraphPad Prism.
   1. In the columns, put the CDNB concentration in mM
   2. In the rows, put the triplicate of the substrate concentration divided by the velocity found during step 10.
   3. Draw the non-linear regression plot.
   4. Km is determined as the negative value of the x intercept. With GraphPad Prism, determine the x intercept.


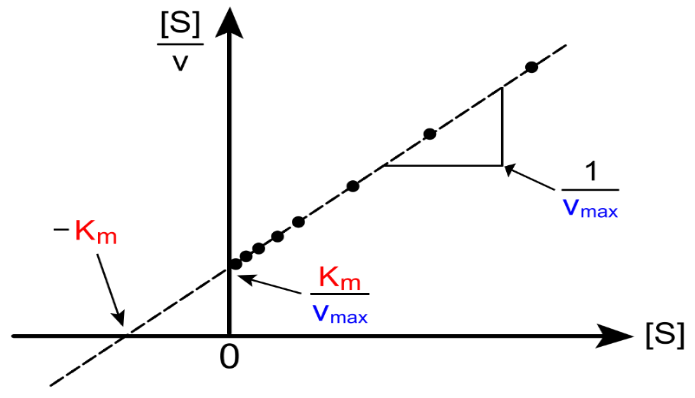


**II. HepaRG treatment viability and GSH assay**

**Solution preparations:**

- Prepare one tube with 200 ul of medium.
- DMSO 10% in medium preparation: 20 ul of DMSO and 180 ul of medium.
- Prepare the following fludarabine solutions:

| **Compound** | **Stock concentration (uM)** | **Final concentration (uM)** | **volume of stock (ul)** | **Volume of solvent (ul)** | **solvent to use** | **Final volume (ul)** | **Dilution factor** | **DMSO concentration (%)** |  |  |
| --- | --- | --- | --- | --- | --- | --- | --- | --- | --- | --- |
| Flu | 100000 | 4000 | 8 | 192 | DMSO | 200 | 25.0 | 100 |  |  |
| Flu | 4000 | 400 | 10 | 90 | DMSO | 100 | 10.0 | 100 |  |  |
| Flu | 4000 | 200 | 5 | 95 | DMSO | 100 | 20.0 | 100 |  |  |
| EA | 25000 | 4000 | 32 | 168 | DMSO | 200 | 6.3 | 100 |  |  |
| EA | 4000 | 400 | 10 | 90 | DMSO | 100 | 10.0 | 100 |  |  |
| EA | 4000 | 200 | 5 | 95 | DMSO | 100 | 20.0 | 100 |  |  |
| Bu | 200000 | 16000 | 16 | 184 | DMSO | 200 | 12.5 | 100 |  |  |
| Bu | 16000 | 1600 | 10 | 90 | DMSO | 100 | 10.0 | 100 |  |  |
| Bu | 16000 | 800 | 5 | 95 | DMSO | 100 | 20.0 | 100 |  |  |
|  |  |  |  |  |  |  |  |  |  |  |
| **Compound** | **Stock concentration (uM)** | **Final concentration (uM)** | **volume of stock (ul)** | **Volume of solvent (ul)** | **solvent to use** | **Final volume (ul)** | **Dilution factor** | **DMSO concentration (%)** | **Final concentration in well (uM)** | **Final DMSO in well %** |
| Flu | 4000 | 400 | 40 | 360 | Medium | 400 | 10 | 10 | 40 | 1 |
| Flu | 400 | 40 | 40 | 360 | Medium | 400 | 10 | 10 | 4 | 1 |
| Flu | 200 | 20 | 40 | 360 | Medium | 400 | 10 | 10 | 2 | 1 |
| EA | 4000 | 400 | 40 | 360 | Medium | 400 | 10 | 10 | 40 | 1 |
| EA | 400 | 40 | 40 | 360 | Medium | 400 | 10 | 10 | 4 | 1 |
| EA | 200 | 20 | 40 | 360 | Medium | 400 | 10 | 10 | 2 | 1 |
| Bu | 16000 | 1600 | 40 | 360 | Medium | 400 | 10 | 10 | 160 | 1 |
| Bu | 1600 | 160 | 40 | 360 | Medium | 400 | 10 | 10 | 16 | 1 |
| Bu | 800 | 80 | 40 | 360 | Medium | 400 | 10 | 10 | 8 | 1 |

**Cell treatment:**

- Add 10 uL medium in “Ctrl” wells
- Add 10 uL of 10% DMSO diluted in medium in the“1% DMSO wells”
- Add 10 uL of the corresponding Flu solution from the second table in the other wells.
- Incubate one plate for 48 hours (GSH) and two plates for 72 hours (GSH and cellTiterglo).

**Plate layout design**

|  | 1 | 2 | 3 | 4 | 5 | 6 | 7 | 8 | 9 | 10 | 11 | 12 |
| --- | --- | --- | --- | --- | --- | --- | --- | --- | --- | --- | --- | --- |
| A |  |  |  |  |  |  |  |  |  |  |  |  |
| B |  | 1% DMSO | EA 2 uM | EA 4 uM | EA 40 uM | Flu 2 uM | Flu 4 uM | Flu 40 uM | Bu 8 uM | Bu 16 uM | Bu 160 uM |  |
| C |  |  |  |  |  |  |  |  |  |  |  |  |
| D |  |  |  |  |  |  |  |  |  |  |  |  |
| E |  | 1% DMSO | EA 2 uM | EA 4 uM | EA 40 uM | Flu 2 uM | Flu 4 uM | Flu 40 uM | Bu 8 uM | Bu 16 uM | Bu 160 uM |  |
| F |  |  |  |  |  |  |  |  |  |  |  |  |
| G |  |  |  |  |  |  |  |  |  |  |  |  |
| H |  |  |  |  |  |  |  |  |  |  |  |  |

**Table 1:** Enzyme kinetics parameter estimation (Km and Vmax) for *GSTA1, GSTM1, GSTP1* isoforms using CDNB as substrate

| **GSTA1** | Michaelis-Menten | | | | |
| --- | --- | --- | --- | --- | --- |
| **Km** |  |  |  |  |  |
| Expt-1 (Experimenter1) | Expt-2 (Experimenter1) | Expt-3(Experimenter2) | Expt-4(Experimenter3) | Stdev | Mean |
| 0.8847 | 0.6017 | 0.6172 | 0.6337 | 0.01 | 0.62 |
| **Vmax** |  |  |  |  |  |
| Expt-1 (Experimenter1) | Expt-2 (Experimenter1) | Expt-3(Experimenter2) | Expt-4(Experimenter3) | Stdev | Mean |
| 360.9 | 363 | 429.9 | 368.8 | 30.26 | 387.23 |
| **Kcat** |  |  |  |  |  |
| Expt-1 (Experimenter1) | Expt-2 (Experimenter1) | Expt-3(Experimenter2) | Expt-4(Experimenter3) | Stdev | Mean |
| 32.5 | 32.7 | 38.7 | 33.2 | 2.72 | 34.85 |
|  |  |  |  |  |  |
|  |  |  |  |  |  |
| **GSTM1** | Michaelis-Menten | | | | |
| **Km** |  |  |  |  |  |
| Expt-1 (Experimenter1) | Expt-2 (Experimenter1) | Expt-3(Experimenter2) | Stdev | Mean | CV |
| 0.6468 | 0.851 | 0.9751 | 0.14 | 0.824 | 16.4 |
| **Vmax** |  |  |  |  |  |
| Expt-1 (Experimenter1) | Expt-2 (Experimenter1) | Expt-3(Experimenter2) | Stdev | Mean | CV |
| 21.15 | 22.01 | 19.25 | 1.15 | 20.80 | 5.5 |
| **Kcat** |  |  |  |  |  |
| Expt-1 (Experimenter1) | Expt-2 (Experimenter1) | Expt-3(Experimenter2) | Stdev | Mean | CV |
| 1.8 | 1.9 | 1.7 | 0.10 | 1.80 | 5.5 |
|  |  |  |  |  |  |
|  |  |  |  |  |  |
| **GSTP1** | Michaelis-Menten | | | | |
| **Km** |  |  |  |  |  |
| Expt-1 (Experimenter1) | Expt-2 (Experimenter1) | Expt-3(Experimenter2) | Stdev | Mean | CV |
| 0.3153 | 0.327 | 0.2413 | 0.038 | 0.295 | 12.9 |
| **Vmax** |  |  |  |  |  |
| Expt-1 (Experimenter1) | Expt-2 (Experimenter1) | Expt-3(Experimenter2) | Stdev | Mean | CV |
| 9.436 | 7.343 | 7.062 | 1.059 | 7.947 | 13.3 |
| **Kcat** |  |  |  |  |  |
| Expt-1 (Experimenter1) | Expt-2 (Experimenter1) | Expt-3(Experimenter2) | Stdev | Mean | CV |
| 0.9 | 0.7 | 0.6 | 0.10 | 0.73 | 13.3 |
