# Supplementary material for: In silico and in vitro investigations of the drug–drug interaction mechanisms between fludarabine and busulfan
Source: Front Pharmacol. 2026 Mar 10;17:1744021. doi: 10.3389/fphar.2026.1744021 (PMC13010162; doi:10.3389/fphar.2026.1744021)
Supplement: Supplementary file 2 [file Supplementaryfile2.ppt]

## Slide 1
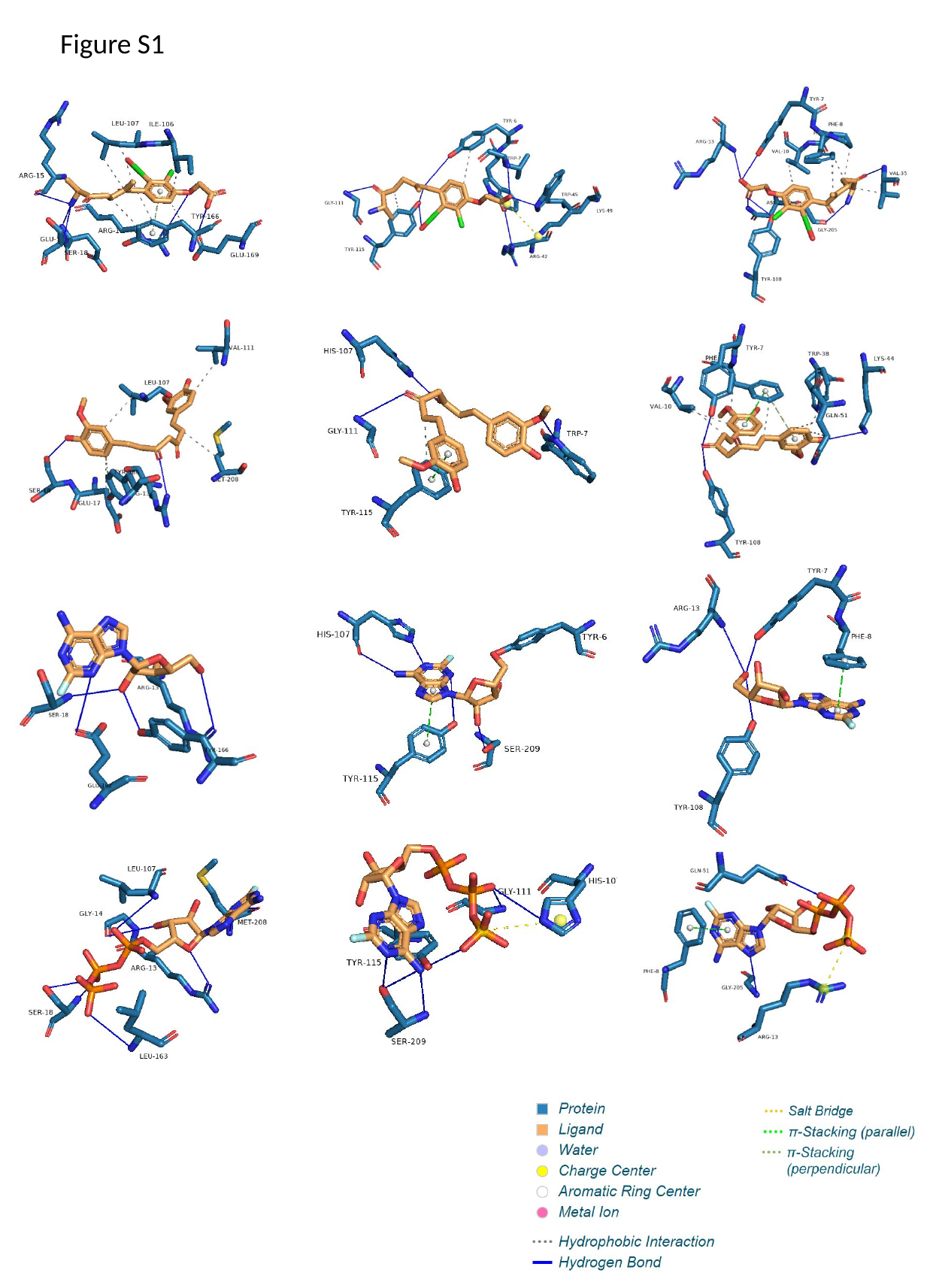

Figure S1

## Slide 2
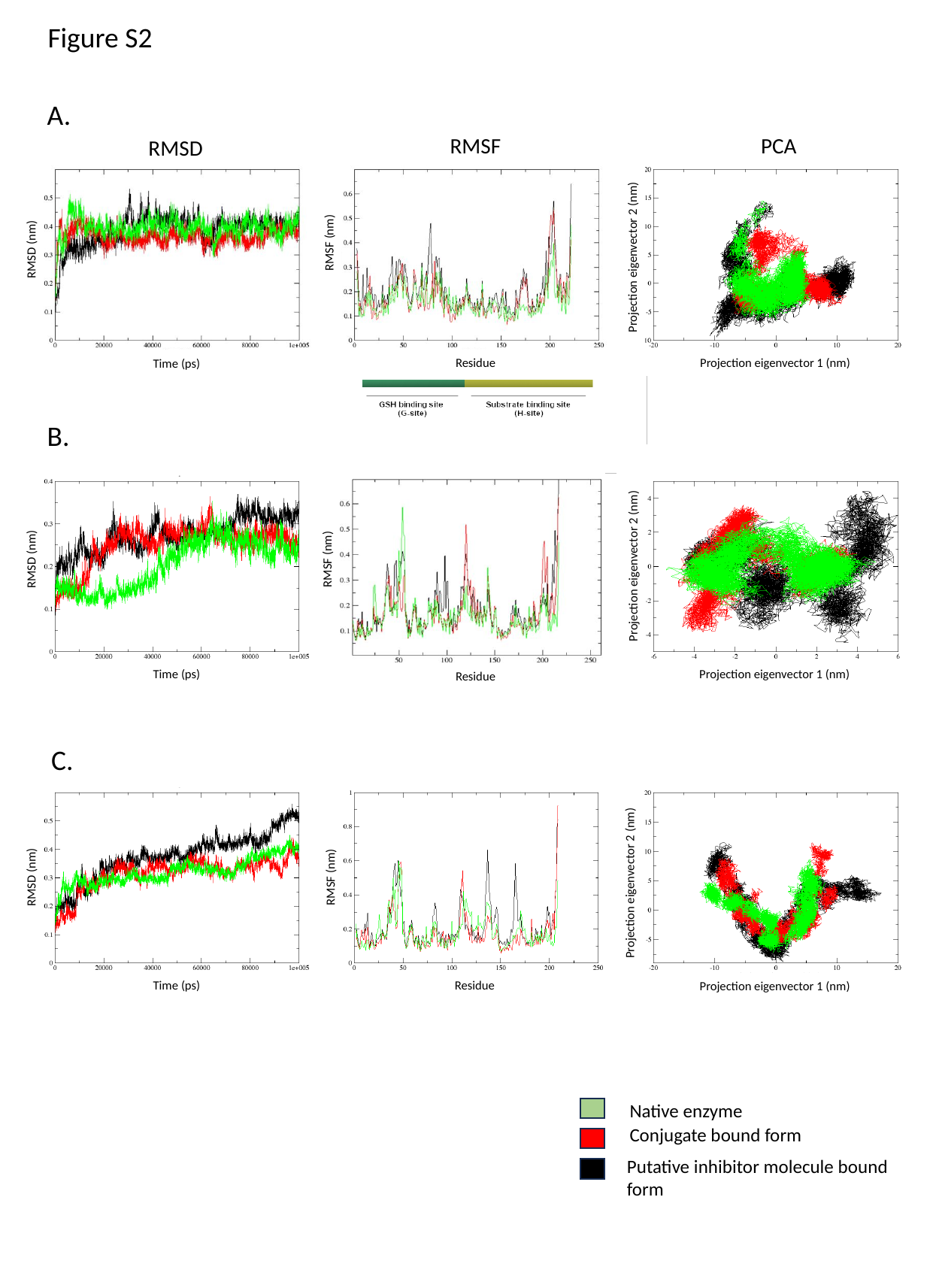

Figure S2
A.
RMSF
PCA
RMSD
RMSF (nm)
RMSD (nm)
Projection eigenvector 2 (nm)
Residue
Projection eigenvector 1 (nm)
Time (ps)
B.
RMSF
RMSF
PCA
RMSD
RMSD (nm)
RMSF (nm)
Projection eigenvector 2 (nm)
Time (ps)
Projection eigenvector 1 (nm)
Residue
C.
PCA
RMSF
RMSD
RMSD (nm)
RMSF (nm)
Projection eigenvector 2 (nm)
Residue
Time (ps)
Projection eigenvector 1 (nm)
Native enzyme
Conjugate bound form
Putative inhibitor molecule bound form

## Slide 3
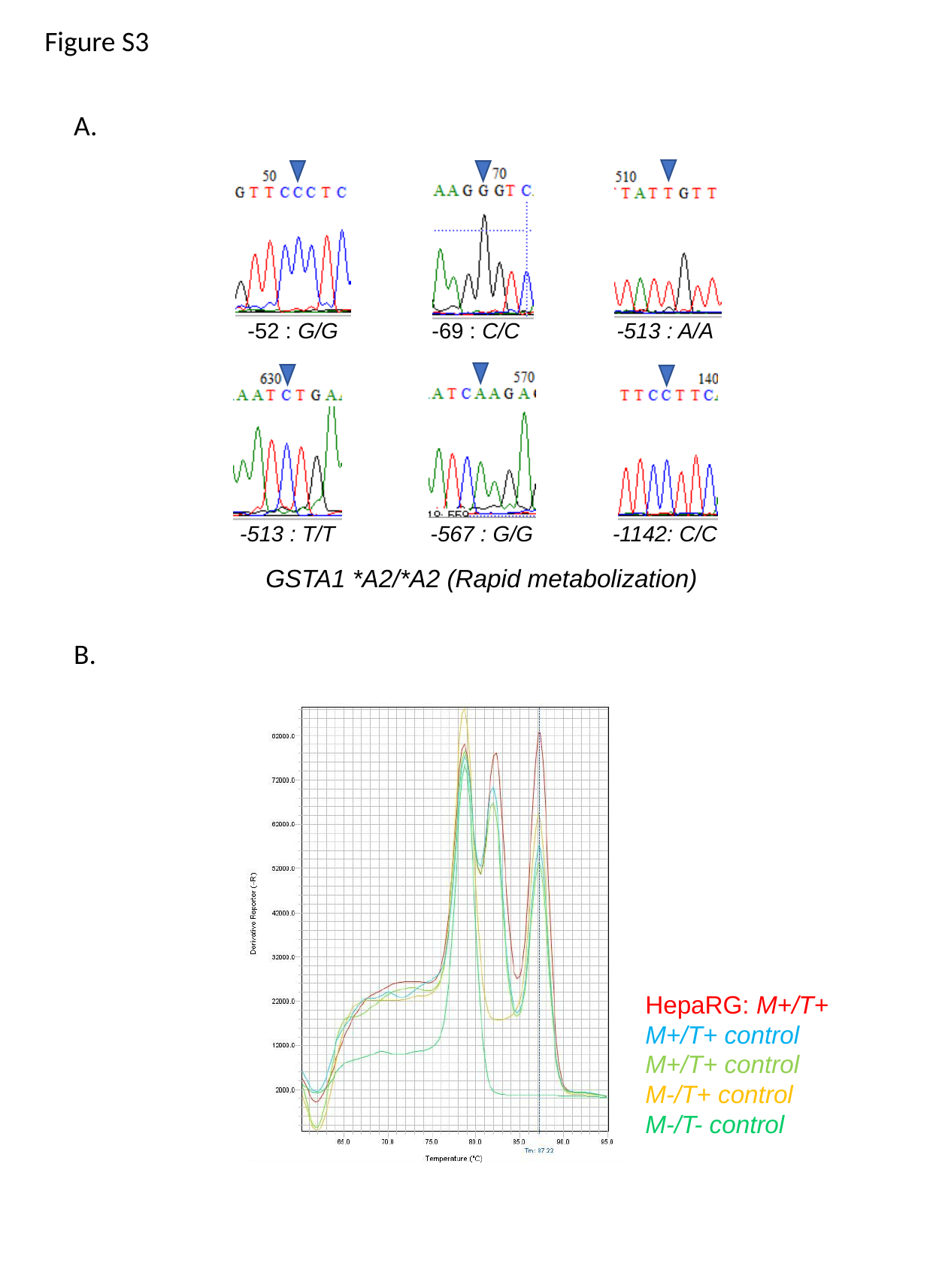

Figure S3
A.
-52 : G/G
-69 : C/C
-513 : A/A
-513 : T/T
-567 : G/G
-1142: C/C
GSTA1 *A2/*A2 (Rapid metabolization)
B.
HepaRG: M+/T+
M+/T+ control
M+/T+ control
M-/T+ control
M-/T- control

## Slide 4
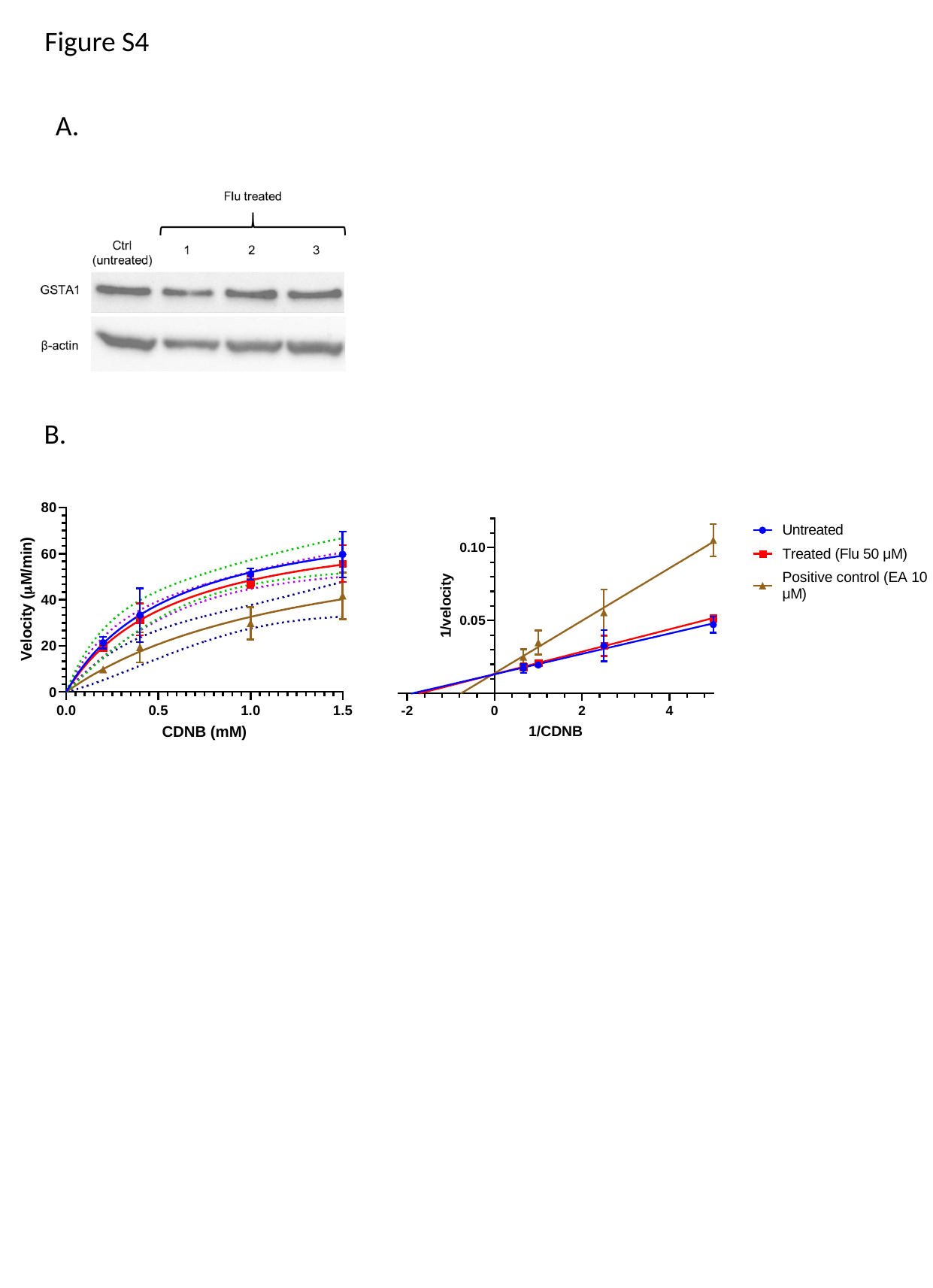

Figure S4
A.
B.
